# Lymph Node Positivity in One-Step Nucleic Acid Amplification is a Prognostic Factor for Postoperative Cancer Recurrence in Patients with Stage II Colorectal Cancer: A Prospective, Multicenter Study

**DOI:** 10.1245/s10434-019-07971-y

**Published:** 2019-11-13

**Authors:** Michio Itabashi, Hirofumi Yamamoto, Naohiro Tomita, Masafumi Inomata, Kohei Murata, Shigeoki Hayashi, Yasuhiro Miyake, Seiji Igarashi, Takeshi Kato, Shingo Noura, Tomohisa Furuhata, Heita Ozawa, Ichiro Takemasa, Masayoshi Yasui, Hiroshi Takeyama, Shu Okamura, Yuko Ohno, Nariaki Matsuura

**Affiliations:** 1 Institute of Gastroenterology, Tokyo Women’s Medical University, Shinjuku, Japan; 2 Department of Molecular Pathology, Division of Health Sciences, Graduate School of Medicine, Osaka University, Suita, Japan; 3 Division of Lower GI Surgery, Department of Surgery, Hyogo College of Medicine, Nishinomiya, Japan; 4 Department of Gastroenterological and Pediatric Surgery, Faculty of Medicine, Oita University, Oita, Japan; 5 Department of Surgery, Kansai Rosai Hospital, Amagasaki, Japan; 6 Department of Digestive Surgery, Nihon University Hospital, Chiyoda, Japan; 7 Department of Surgery, Osaka Minato Central Hospital, Osaka, Japan; 8 Division of Pathology, Tsuboi Cancer Center Hospital, Koriyama, Japan; 9 Department of Colorectal Surgery, National Hospital Organization Osaka National Hospital, Osaka, Japan; 10 Department of Surgery, Osaka Rosai Hospital, Sakai, Japan; 11 Division of Gastroenterological and General Surgery, St. Marianna University Toyoko Hospital, Kawasaki, Japan; 12 Department of Colorectal Surgery, Tochigi Cancer Center, Utsunomiya, Japan; 13 Department of Surgery, Surgical Oncology and Science, Sapporo Medical University, Sapporo, Japan; 14 Department of Gastroenterological Surgery, Osaka International Cancer Institute, Osaka, Japan; 15 Department of Surgery, Minoh City Hospital, Minoh, Japan; 16 Department of Surgery, Suita Municipal Hospital, Suita, Japan; 17 Department of Mathematical Health Science, Graduate School of Medicine, Osaka University, Suita, Japan; 18 Osaka International Cancer Institute, Osaka, Japan

## Abstract

**Background:**

For colorectal cancer (CRC) patients, the standard histological lymph node (LN) evaluation has low sensitivity. Our previously developed one-step nucleic acid amplification (OSNA™) assay measures cytokeratin 19 gene expression in whole LNs. We recently showed that 17.6% of pN0 stage II CRC patients were OSNA positive, suggesting a correlation between OSNA results and disease recurrence. This multicenter, prospective study investigateed the prognostic value of the OSNA assay for pStage II CRC patients.

**Methods:**

We examined 204 CRC patients who were preoperatively diagnosed as cN0 and cN1 and surgically treated at 11 medical institutions across Japan. Nine patients were excluded, and 195 patients (Stage I: *n* = 50, Stage II: *n* = 70, Stage III: *n* = 75) were examined. All LNs, harvested from patients, were examined histopathologically using one-slice hematoxylin–eosin staining. Furthermore, half of the LNs was examined by the OSNA assay. Patients were classified according to the UICC staging criteria and OSNA results, and the 3-year, disease-free survival (DFS) of each cohort was analyzed.

**Results:**

Average 21.2 LNs/patient were subject to pathological examination. Approximately half of all harvested LNs (average, 9.4 LNs/patient) were suitable for the OSNA assay. Significantly lower 3-year DFS rates were observed in pStage (pathological Stage) II OSNA-positive patients than in OSNA-negative patients (*p* = 0.005). Among all assessed clinical and pathological parameters, only the OSNA result significantly affected 3-year DFS rates in pStage II CRC patients (*p* = 0.027).

**Conclusions:**

This study shows that OSNA positivity is a risk factor for recurrence of the patients with pStage II CRC.

**Electronic supplementary material:**

The online version of this article (10.1245/s10434-019-07971-y) contains supplementary material, which is available to authorized users.

Patients with stages I–II (pN0) colorectal cancer (CRC) with no high-risk factors are usually treated by surgical resection and avoid adjuvant chemotherapy, which is only recommended for patients with high-risk stage II and stage III disease, due to significant side effects.^1^^,^^2^ The standard histopathological evaluation of lymph nodes (LNs) in patients with CRC is based on hematoxylin and eosin (H&E) staining of LNs and usually involves only approximately 5% of LN tissues (2–5-µm-thick LN sections). Therefore, the sensitivity of the LN analysis often is low, leading to disease recurrence in up to 25% of patients with pN0 disease.^3^^–^6

Accumulating evidence supports the usefulness of molecular methods for identifying micrometastases and enhancing the sensitivity of pathological cancer staging.^7^ One of these molecular methods, the one-step nucleic acid amplification (OSNA™) assay, was first introduced by Tsujimoto et al.^8^ for detecting LN metastases in patients with breast cancer. The authors demonstrated a 98.2% agreement rate between OSNA results and routine histopathological findings from the same LN, with practically no false-positive results. Subsequent studies have further demonstrated the high sensitivity of OSNA in detecting LN metastasis of breast, gastric, lung, and colorectal cancers.^9^^–^16 Our own previous prospective, multicenter studies conducted in Japan demonstrated the usefulness of the OSNA assay as a complementary tool for diagnosing LN metastasis and CRC staging.^17^^,^^18^ The later study revealed that 17.6% (13/74) of patients with histologically node-negative stage II CRC were nodal positive by the OSNA assay.^18^ Based on this observation, we hypothesized that positive results of the OSNA assay in patients with node-negative pStage II CRC would correlate with poor disease prognosis. To investigate the prognostic value of the OSNA assay in patients with pStage II CRC, we conducted this multicenter trial.

## Materials and Methods

### Patients

This prospective, multicenter study conducted in Japan was approved by the institutional review board of Tokyo Women’s Medical University (approval number: 4267). The University Medical Information Network of Japan registration number of the study is UMIN000023233. In addition, ethical approval was obtained from each participating institution. Informed consent was obtained from all patients before enrollment, and patients who were treated with neoadjuvant therapy before surgery, had distant metastases, or had any previous or current cancer were excluded.

A total of 204 patients with CRC who were preoperatively diagnosed as having cN0 and cN1 cancer using a computed tomography or magnetic resonance imaging scan and who were surgically treated at 11 medical institutions across Japan between May 2012 and August 2013 were recruited for our study. An open complete mesocolic excision or laparoscopic surgery was performed for tumor resection. LNs were harvested from tissue specimens immediately after surgery using a standardized study protocol.^19^ The Union for International Cancer Control classification was used for patient staging. Four patients with pStage IV CRC, four with follow-up durations < 50 days, and one with no follow-up data were excluded. Among the remaining 195 patients, 50 were diagnosed with pStage I CRC, 70 with pStage II CRC, and 75 with pStage III CRC.

### Study Design

**Fig. 1:**
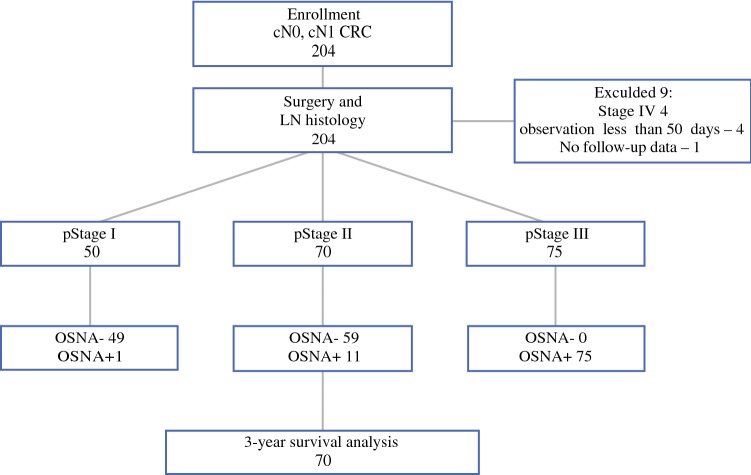
Flowchart of this study’s experimental design

This study was designed to assess the clinical significance of OSNA positivity in patients with pStage II CRC, and survival rates of 70 patients with pStage II CRC were analyzed. The flow chart of the study design is shown in Fig. 1. The decision to administer adjuvant treatment was solely based on the results of the standard histopathological examination and not on the OSNA results.

### LN Processing and Examination

Immediately after surgery, LNs were harvested from fresh biopsy specimens. When the long axis was ≥ 4 mm, LNs were divided into two pieces, and one piece was used for the OSNA assay and the other for standard histology (one slice H&E staining). Small LNs that did not have sufficient tissue to divide into two pieces (i.e., < 4 mm) were only examined by histopathology. Thus, all of the LNs were examined by standard histology (average 21.2, median 19.0 LNs/patient, range 1–75), and average 9.4, median 8.0 LNs/patient (range 2–25) were examined by the OSNA assay. The LN tissues used for OSNA assays were frozen and stored at − 80 °C until the assay was performed.

### OSNA Assay

The OSNA assay has been previously described.^17^^,^^18^ Briefly, LNs were homogenized with 4 mL of a lysis buffer solution (Lynorhag; Sysmex, Kobe, Japan) after surgically removing the extranodal tissue and centrifuged at 10,000×*g*. A 2-µL sample of the supernatant was analyzed using the RD-100i system (Sysmex), which is an automated gene amplification detection system, using a reverse transcription loop-mediated isothermal amplification method and with the Lynoamp BC kit (Sysmex). Pyrophosphate, a byproduct of the reaction, indicated the degree of amplification. The precipitation of magnesium pyrophosphate caused a change in turbidity, correlating with the CK19 mRNA copy number in the original lysate. A preestablished standard curve of three calibrators that contained different CK19 mRNA copy numbers was used in the assay to calculate CK19 mRNA copy numbers. For each assay, standard positive and negative control samples were used for quality assurance. LNs containing > 250 mRNA copies/µL were considered OSNA positive as previously described.^17^

### Statistical Analysis

Postoperative overall survival (OS) and disease-free survival (DFS) curves were produced using the Kaplan–Meier method. OS was measured from the date of surgery to the date of death or the last follow-up examination. To evaluate the significance of differences in survival rates among the groups, a log-rank test was performed. A univariate analysis with the Cox proportional hazards model was used to establish associations between OSNA assay results and other histopathological parameters. All statistical comparisons were conducted using the *χ*^2^ test. *P* values < 0.05 were considered statistically significant. All statistical analyses were conducted using Prism Mac version 5.0b (graphpad.com) and JMP Pro version 13.1.0 (SAS Institute Inc.) software.

## Results

### OSNA-Based Upstaging

When OSNA assay results were combined with TNM standard histopathological staging (single-section H&E staining) for 204 patients with cN0 and cN1 CRC, most of the upstaging occurred for patients with pStage II, of whom 11 of 70 patients (15.7%) were OSNA positive (Table 1). OSNA-positive LNs were found mostly in the paracolic or pararectal LNs near the primary tumor in 10 of 11 OSNA-positive Stage II CRC patients. A comparison of demographic and histopathological characteristics of patients who were OSNA negative and OSNA positive is shown in Supplementary Table 1. For 22 of 70 patients (31%), the number of harvested LNs was < 12. Postoperative adjuvant chemotherapy (Supplementary Table 2) was performed for 19 patients (27%) based only on histological data and the principal physician’s decision (OSNA-positive group 55%, OSNA negative group 22%). The median follow-up period was 48.5 months (range: 1.7–53.4 months).

**Table 1:** Staging by histopathological examinations with OSNA evaluations

	OSNA + pathology				Upstage rate %
	I	II	III	Total	
*Pathology*					
I	49	0	1	50	2.0
II	0	59	11	70	15.7
III	0	0	75	75	–
Total	49	59	87	195	–

### DFS and OS of OSNA-Negative and OSNA-Positive Cohorts in pStage II CRC

**Fig. 2:**
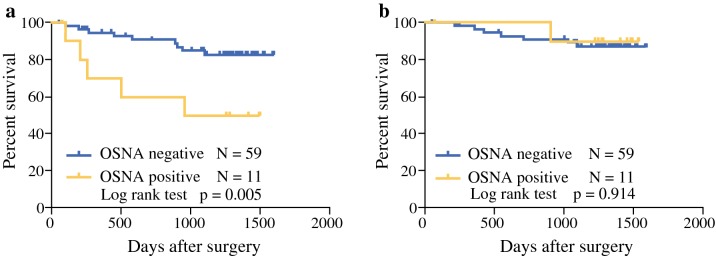
Survival rates of OSNA-positive and OSNA-negative patients in pStage II CRC patients. **a** 3-year disease-free survival rate and **b** 3-year overall survival rate

In pStage II CRC, significantly higher 3-year DFS rates were observed in OSNA-negative patients than in OSNA-positive patients (86% vs. 55%; *p* = 0.005; Fig. 2a), whereas 3-year OS did not significantly differ between OSNA-negative and OSNA-positive patients (*p* = 0.914; Fig. 2b).

### Univariate Analysis of OSNA Assay Results

A univariate analysis of OSNA positivity confirmed its significance as a recurrence risk factor for patients with pStage II CRC (*p* = 0.027; Table 2). Other demographic and histopathological parameters, including age, sex, tumor location, tumor size, vascular and lymphatic invasion, and other features, were not risk factors for tumor recurrence in patients with pStage II CRC (Table 2).

**Table 2:** Univariate analysis of the OSNA results

	Hazard ratio	95% CI	*p* value
OSNA status in LNs [(+) vs. (−)]	3.856	1.184–11.178	0.027
Age (> 70 vs. ≤ 70 years)	1.711	0.594–5.202	0.317
Sex (male vs. female)	1.774	0.613–5.773	0.294
Tumor location (rectum vs. colon)	1.737	0.393–5.569	0.423
Colon tumor location (left vs. right)	2.238	0.675–8.556	0.189
Tumor size (≤ 4.5 vs. > 4.5 cm)	1.22	0.424–3.706	0.712
Differentiation (well^a^, moderate^b^ vs. poor^c^, muc^d^, others)	5.50E + 08	0.555–0.555	0.138
Lymphatic invasion (present vs. absent)	1.954	0.675–6.360	0.219
Venous invasion (present vs. absent)	1.008	0.350–3.061	0.989
T stage (T4 vs. T3)	2.302	0.631–6.885	0.188
Retrieved lymph node number (< 12 vs. ≥ 12)	2.161	0.709–6.239	0.167

^a^Well indicates a well-differentiated adenocarcinoma

^b^Moderate indicates a moderately differentiated adenocarcinoma

^c^Poor indicates a poorly differentiated adenocarcinoma

^d^Muc indicates a mucinous adenocarcinoma

The histopathological characteristics of five recurrent tumors in OSNA-positive patients are presented in Table 3. The site of metastases and number of OSNA mRNA copies per microliter varied. No associations were observed among recurrent site, primary tumor wall invasion depth, and primary tumor site. Standard adjuvant chemotherapy with oral anticancer agents was administered to four of five patients, and only one patient with OSNA-positive recurrence had < 12 LNs resected and analyzed.

**Table 3:** Recurrences in patients with OSNA-positive pStage II CRC

	Recurrence	PT	Location	Number of harvested LNs	OSNA (copies/uL)	Histology		Adjuvant chemotherapy
Case 1	Lung	T3	S	20	350	Moderate*	Yes	Tegafur/uracil plus leucovorin (UFT/LV)
Case 2	Liver	T3	RS	9	1600	Moderate	Yes	Tegafur/uracil plus leucovorin (UFT/LV)
Case 3	Lung	T3	A	25	8600	Moderate	Yes	Tegafur/uracil plus leucovorin (UFT/LV)
Case 4	Local	T4b	T	26	2500	Moderate	No	–
Case 5	Lung	T3	A	14	1100	Moderate	Yes	Tegafur/gimeracil/teracil (S-1)

*Moderate indicates a moderately differentiated adenocarcinoma

## Discussion

The prediction of tumor recurrence after resection in patients with pStage II CRC is challenging.^20^^,^^21^ This prospective, multicenter study confirmed previous observations of the presence of undetected nodal micrometastases that were identified by the OSNA assay in patients with pStage II CRC and demonstrated that nodal positivity in the OSNA assay was an objective risk factor in these patients.^22^^,^^23^ We found that 15.7% of pStage II CRC patients were OSNA-positive. Other studies also showed that pN0 CRC patients were up-staged by OSNA test in 15.3–28.8%.^13^^,^^15^^,^^24^^,^^25^ It is suggested that difference of detection rate depends on sampling of LN volume and number.^24^ Notably Rakislova et al.^24^ reported that 20.8% pN0 cases were OSNA-positive by the pooling method which would be helpful as compared to time consuming and expensive individual analysis of LNs.

Previous similar studies were unable to assess the prognostic value of the molecular detection of CK19 mRNA copies in LNs of patients with pStage II CRC because of the limited 2-year observation time.^26^ In this study, we clearly demonstrated the correlation between molecular LN status and 3-year DFS rates. Furthermore, we showed that the 3-year DFS of patients with positive OSNA results was significantly lower than that of those with negative OSNA results. However, no difference in OS was found between these groups, most likely because OS assessment requires a longer follow-up period and larger sample of patients who are OSNA-positive.

Yamamoto et al.^27^ reported that the 5-year OS and DFS of patients with higher CEA (carcinoembryonic antigen) mRNA expression levels, top 30% of micrometastases in pStage II CRC, were significantly low, and the volume of micrometastases was related to the prognosis of the pStage II CRC. Compared with the findings of Yamamoto et al.’s study, only 15.7% of pStage II CRC were molecular positive in OSNA assay in our study. The OSNA assay can discriminate macrometastases from micrometastases by assessing metastasis volume.^8^ Therefore, metastasis volume of OSNA positivity in our study would be much higher than that in Yamamoto et al.’s study, demonstrating that the OSNA status is related to the DFS of pStage II CRC.

According to the univariate analysis, cancer recurrence correlated only with the OSNA status (*p* = 0.027), while other parameters were not significant in this study. The guidelines of ASCO, ESMO, or NCCN recommend different risk factors for stage II CRC among the parameters, such as vascular invasion, lymphatic invasion, T4, obstruction, perforation, < 12 LNs examined, poorly or undifferentiated histology, high serum CEA and others, but the evidence level of these factors is not high.^28^^–^30 Considering that lymph node metastasis is a well-established predictive marker for survival in stage III CRC patients, it is likely that high metastasis volume detected by OSNA test may predict pStage II patients’ survival as well.

Recently Wild et al.^31^ performed a systematic review and meta-analysis of OSNA use in CRC and concluded that long-term outcomes and the value of adjuvant therapy in those upstaged by OSNA should be clarified before routine use of OSNA test. In this regard, our study may be constructive because it firstly showed significantly worse 3-year DFS in OSNA-positive pStage II group. However, we should emphasize that this study has limitation of small sample size, which may be associated with relatively high relapse rate of stage II patient compared with the large-scale study.^32^ Another limitation is that 22 of 70 stage II (31.4%) CRC patients had < 12 LNs examined. We cannot deny a possibility that a portion of them might be at stage III if ≥ 12 LNs had been examined although resected LN numbers were not significantly different between OSNA(+) and OSNA(−) group in pStage II CRC patients.

Because the OSNA test is at present covered by insurance in Japan for diagnosis of LN metastasis of CRC, cumulative data in clinics would lead to firm conclusion in near future. It is well established that the presence of LN metastasis is a clear indication for adjuvant chemotherapy after cancer resection.^30^ Our results support the idea that patients with pN0 OSNA-positive CRC might also need chemotherapy after curative surgery. To achieve this goal, we are currently underway to conduct the observational research in which the recurrence rate will be compared between the groups of no treatment or adjuvant chemotherapy after surgery both in OSNA-positive pStage II CRC patients. The result would clarify whether adjuvant chemotherapy is beneficial to patients with OSNA-positive pStage II CRC.

## Conclusions

This prospective, multicenter study is the first to assess the prognostic value of OSNA positivity in patients with pStage II CRC, which serves as a marker of high recurrence risk. Further studies are warranted to optimize treatment strategies in this setting.

## Electronic supplementary material

Below is the link to the electronic supplementary material.

Supplementary material 1 (DOCX 125 kb)
