# Supplementary material for: Lymph Node Positivity in One-Step Nucleic Acid Amplification is a Prognostic Factor for Postoperative Cancer Recurrence in Patients with Stage II Colorectal Cancer: A Prospective, Multicenter Study
Source: Ann Surg Oncol. 2019 Nov 13;27(4):1077–83. doi: 10.1245/s10434-019-07971-y (PMC7060165; doi:10.1245/s10434-019-07971-y)
Supplement: Supplementary file 1 — Supplementary material 1 (DOCX 125 kb) [file 10434_2019_7971_MOESM1_ESM.docx]

**SUPPLEMENTARY TABLE 1**  Characteristics of patients with pStage II CRC

*

*Well indicates a well differentiated adenocarcinoma;

Mod indicates a moderately differentiated adenocarcinoma;

Poor indicates a poorly differentiated adenocarcinoma;

Muc indicates a mucinous adenocarcinoma.

**SUPPLEMENTARY TABLE 2**

Adjuvant chemotherapies of patients with pStage II CRC

*CapeOX: Capecitabine + Oxaliplatin
